# Sex-specific outcomes in methotrexate therapy among patients with psoriatic arthritis and psoriasis

**DOI:** 10.1016/j.ero.2026.100255

**Published:** 2026-10-10

**Authors:** Liana Barenbrug, Gonul Hazal Koc, Marisol E Otero, Emmerik F A Leijten, Sander I van Leuven, Irene E van der Horst-Bruinsma, Juul M P A van den Reek, Elke M G J de Jong, Marc R Kok, Marijn Vis, Michelle L M Mulder

**Affiliations:** a Department of Dermatology, Radboud University Medical Center, Nijmegen, The Netherlands; b Department of Rheumatology, Erasmus Medical Center, Rotterdam, The Netherlands; c Department of Rheumatology, Sint Maartenskliniek, Nijmegen, The Netherlands; d Department of Rheumatology, Radboud University Medical Center, Nijmegen, The Netherlands; e Department of Rheumatology and Clinical Immunology, Maasstad Hospital, Rotterdam, The Netherlands

## Abstract

**Objectives:**

The purpose of this paper is to identify sex-specific patterns in methotrexate (MTX) therapy among patients with psoriatic arthritis (PsA) and psoriasis (PsO).

**Methods:**

Data from adult patients treated with MTX monotherapy included in the Dutch southwest Early Psoriatic Arthritis Registry (rheumatology) or Continuous Assessment of Psoriasis Treatment Use REgistry with Methotrexate (dermatology) registries were obtained to create 2 cohorts of patients with PsA or PsO, respectively. Drug survival was assessed using Kaplan-Meier and Cox regression analyses. Disease activity, dosing, adverse events, and subsequent treatments were evaluated.

**Results:**

A total of 525 (50.1% female) patients were included in the PsA cohort and 217 (41.1% female) patients in the PsO cohort. In the PsA cohort, female sex was associated with shorter drug survival (adjusted hazard ratio [HR] = 2.5; 95% CI, 1.6-4.1; *P* < .001), whereas in the PsO cohort, female sex was associated with longer drug survival (HR = 0.6; 95% CI, 0.4-0.9; *P* = .008). Disease activity at MTX discontinuation was higher in females than males in the PsA cohort (Psoriatic Arthritis Disease Activity Score: 3.9 [1.0] vs 3.1 [1.5], *P* = 0.037), whereas in the PsO cohort, disease activity was higher in males than females (Psoriasis Area and Severity Index: 4.8 [6.4] vs 3.5 [3.7], *P* = .032). MTX dosing was comparable between sexes, though average doses were higher in the PsA than in the PsO cohort (18.2 [7.5] vs 15.0 [3.3] mg/wk, *P* < .001).

**Conclusions:**

Drug survival and treatment response of MTX vary between PsA and PsO, with females in the PsA cohort while males in the PsO cohort having worse outcomes.

## Graphical abstract

**Figure:**
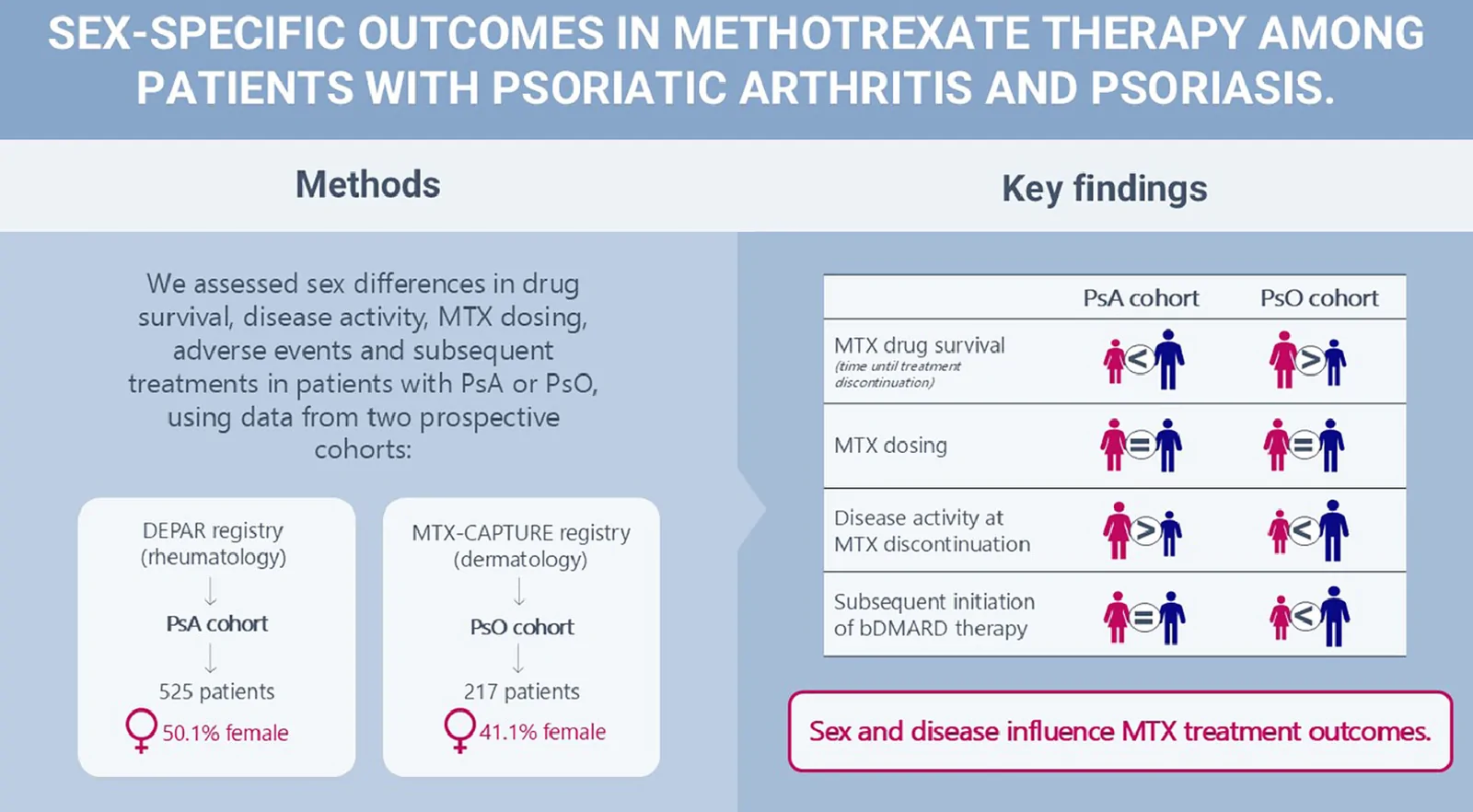

**WHAT IS ALREADY KNOWN ON THIS TOPIC**

• Previous studies on sex differences in the treatment of psoriatic arthritis (PsA) and psoriasis (PsO) have mainly focused on biological disease-modifying antirheumatic drug therapy.
• Knowledge of sex differences in the treatment with methotrexate (MTX) remains limited, despite its wide use as first-line systemic therapy for both PsA and PsO.

**WHAT THIS STUDY ADDS**

• Sex-specific patterns in drug survival differ for patients with PsA and PsO: females with PsA have worse MTX drug survival, whereas males with PsO have worse drug survival.
• Severe disease and adverse events in male patients with PsO may influence distinct drug survival patterns.

**HOW THIS STUDY MIGHT AFFECT RESEARCH, PRACTICE OR POLICY**

• Both sex- and disease-specific patterns should be considered to optimise and individualise MTX therapy in PsA and PsO.

Alt-text: Unlabelled box dummy alt text

## INTRODUCTION

Psoriasis (PsO) is a common chronic inflammatory skin disease affecting 2% to 3% of the population in industrialised countries, with up to 30% of these patients developing psoriatic arthritis (PsA) [1,2]. The prevalence of both PsA and PsO is comparable between sexes; however, prior studies have identified sex differences in disease severity and clinical presentation [[2], [3], [4]]. For example, in patients with PsA, females showed higher disease activity in terms of laboratory inflammatory parameters and joint counts, whereas males tended to have more skin involvement [5,6]. Among patients with PsO, males also showed more severe skin involvement compared with females at the initiation of biologic therapies [[7], [8], [9]]. In recent years, sex differences in the treatment of PsA and PsO, particularly with biological disease-modifying antirheumatic drugs (bDMARDs) have gained increasing attention. Females with PsA and PsO are more likely to discontinue bDMARD therapy than males, with adverse events being a more frequent reason for discontinuation among females [[9], [10], [11], [12], [13], [14]]. In contrast, evidence on sex-related differences in the use of conventional synthetic DMARDs (csDMARDs), such as methotrexate (MTX), remains limited [5,15]. Given that MTX is still being widely used as first-line systemic treatment for both PsA and PsO, a better understanding of potential sex differences is important, as it could support clinicians in making more informed and personalised treatment decisions [[16], [17], [18], [19]]. Therefore, this study aims to further investigate sex differences in MTX drug survival and related treatment outcomes in patients with PsA and PsO.

## PATIENTS AND METHODS

### Patients and setting

Data from adult patients diagnosed with PsA or PsO who were treated with MTX between August 2013 and March 2025 in the DEPAR (Dutch southwest Early Psoriatic Arthritis Registry) or MTX-CAPTURE (Continuous Assessment of Psoriasis Treatment Use REgistry with Methotrexate) registry were extracted. The DEPAR is a multicentre (n = 14), prospective cohort including newly diagnosed DMARD-naïve patients with PsA receiving usual care across the south-west of the Netherlands [20]. The MTX-CAPTURE registry is a prospective observational daily practice registry containing information about adult patients with plaque PsO who are treated with MTX at the outpatient clinic of the Department of Dermatology at the Radboud University Medical Center (Radboudumc) in Nijmegen, the Netherlands [15]. Patients being treated with MTX monotherapy (either oral or subcutaneous) for PsA/PsO were included; consequently, patients who concomitantly used other csDMARDs or bDMARDs at MTX initiation were excluded. MTX monotherapy was generally provided in combination with 10 mg of folic acid per week (5 mg twice weekly) in both the DEPAR and MTX-CAPTURE cohorts.

All PsA and PsO diagnoses were confirmed by a rheumatologist or dermatologist, respectively. In the MTX-CAPTURE registry, patients with PsO were routinely screened for PsA. For this study, patients from the DEPAR registry were included in the PsA cohort, with the rheumatologist as prescribing physician, and patients from the MTX-CAPTURE registry were included in the PsO cohort, with the dermatologist as prescribing physician. Accordingly, patients with PsO in the MTX-CAPTURE registry with a concomitant PsA diagnosis at baseline or during MTX treatment were included in the PsO cohort, as MTX was primarily prescribed for their skin disease.

Ethical approval was obtained for the DEPAR registry (file number: MEC-2012-549). The MTX-CAPTURE registry was reviewed by the Dutch medical research ethics committee Oost-Nederland and exempted for ethical approval according to the Dutch Medical Research Involving Human Subjects Act (file number: 2020-7151). All patients provided informed consent.

### Data collection

To assess sex differences in patient characteristics and MTX treatment outcomes at baseline and at the time of MTX discontinuation, the following data were collected: sex, age at disease onset, disease duration until the start of MTX treatment, age at the start of MTX treatment, body mass index (BMI), Psoriasis Area and Severity Index (PASI) scores (range 0-72, with a higher score indicating more severe disease). Biological sex was recorded as documented in the electronic patient records, which was based on patient-reported information and official identification documents. For patients in the DEPAR registry, data on Psoriatic Arthritis Disease Activity Scores (PASDAS) (range 0-10, with a higher score indicating more severe disease), Swollen Joint Count of 66 Joints, Tender Joint Count of 68 Joints, Leeds Enthesitis Index, Dactylitis count, C-reactive protein levels, Health Assessment Questionnaire, Physician Global Visual Analogue Scale (VAS), Patient VAS, Physical Component Summary Short Form 36 were also extracted. In the DEPAR registry, reasons for MTX discontinuation were classified as ‘adverse events’, ‘ineffectiveness’, ‘adverse events and ineffectiveness’, ‘remission’, ‘death’, ‘other’, or ‘unknown’. The MTX-CAPTURE registry used the same classification scheme, but further specified the ‘other’ category into ‘pregnancy wish’ and ‘other’. Dosages of MTX were standardised and expressed in milligrams per week. Adverse events that led to MTX discontinuation were extracted from the MTX-CAPTURE registry and categorised using Medical Dictionary for Regulatory Activities version 27 but were not available in the DEPAR cohort. For patients who discontinued MTX, subsequent initiation of bDMARD therapy within 90 days was assessed.

### Statistical analyses

All analyses were performed in SPSS version 29.0 (IBM Corporation). Baseline patient and treatment characteristics were displayed as means with SD for normally distributed continuous variables, medians with IQR for skewed continuous variables, and numbers with percentages for categorical variables. To compare patient groups, an independent t test was used for continuous variables with a normal distribution, Mann-Whitney U test for continuous variables with a skewed distribution, and N-1 Chi-squared test for categorical variables. Comparisons of patient characteristics at the start of and discontinuation of MTX between males and females were considered exploratory and intended to describe potential sex-related differences rather than to formally test multiple prespecified hypotheses. Therefore, no formal adjustment for multiple testing was applied. The reported *P* values should be interpreted with appropriate caution.

For drug survival analyses, a treatment episode was defined as a continuous period during which a patient received MTX monotherapy without an interruption exceeding 90 days. In cases where a patient had >1 treatment episode of MTX, only the first episode was included in the analysis. Drug survival rates were estimated using Kaplan-Meier analysis. The overall drug survival curve incorporated all causes of treatment discontinuation as events, including ineffectiveness, adverse events, and death. Switching to or concomitantly initiating a bDMARD was considered discontinuation due to ineffectiveness. Patients were censored when lost to follow-up or if still using MTX at data lock. Additionally, separate drug survival analyses were conducted for discontinuation due to ineffectiveness and adverse events. For these cause-specific analyses, patients were also censored if they discontinued MTX for a reason other than the reason of interest (ie, discontinuation due to remission). The log-rank test was used to compare survival curves between sexes. In addition, we conducted drug survival analyses in which sex groups were further stratified based on age (young adults [≥18 and ≤30 years] vs adults [>30 years]). To assess the impact of concomitant PsA in the PsO cohort, a sensitivity analysis was conducted excluding patients with PsA from the PsO cohort. Kaplan-Meier curves were visualised using R Studio (Posit) version 2024.04.

Cox regression models adjusted for precision variables (ie, variables independent of the exposure variable but predictive of the outcome) were used to examine the association of sex with drug survival. Precision variables included age, disease duration, BMI, PsA diagnosis, and disease activity (measured by PASDAS for PsA and PASI for PsO). All precision variables were assessed at baseline (ie, the start of the MTX treatment episode of interest). Model assumptions were checked with residual plots. Missing data for the precision variables used for the adjusted model (disease duration, BMI, type of PsA, baseline PASDAS, and baseline PASI) were imputed using multiple imputation. Imputed variables were created and pooled in the model 10 times.

## RESULTS

### Patient characteristics

This study included 525 patients in the PsA cohort and 217 in the PsO cohort, of which 50.1% and 41.1% were females, respectively (Table 1). The total cumulative actively treated observation period for the PsA cohort was 1073.7 years (males [M]: 654.6 years vs females [F]: 419.1 years), and 648.8 years for the PsO cohort (M: 364.0 years vs F: 284.8 years). Patients in the PsO cohort were diagnosed at a younger age (28.0 [25.0]) compared with those in the PsA (53.0 [20.8]) cohort (*P* < .001), whereas age at MTX initiation was similar between groups. As a result, disease duration before MTX initiation was substantially longer in the PsO cohort (13.9 years [18.4]) than for the PsA cohort (0.04 years [0.1]) (*P* < .001). The PASI score was higher in the PsO cohort compared with patients in the PsA cohort (7.3 [5.9] vs 2.0 [4.2]), *P* < .001). Baseline PASDAS scores in the PsA cohort (M: 4.3 [1.2] vs F:4.4 [1.1], *P* = .402) and baseline PASI scores in the PsO cohort (M: 6.9 [7.5] vs F:7.8 [4.8], *P* = .377) were comparable among female and male patients. Males in the PsA cohort had a higher baseline PASI score compared with females (M: 2.5 [5.0] vs F: 1.0 [3.4], *P* < .001).

**Table 1:** Baseline characteristics of patients being treated with methotrexate in the psoriatic arthritis cohort and psoriasis cohort

Characteristic	PsA cohort				PsO cohort				
	Total	Males	Females	*P* value (M vs F)	Total	Males	Females	*P* value (M vs F)	*P* value (PsA vs PsO)
No. of patients	525	262 (49.9%)	263 (50.1%)	-	217	128 (59.0%)	89 (41.0%)	-	-
Age at diagnosis (y)	53.0 [20.8]	53.9 [19.3]	52.0 [23.0]	.298	28.0 [25.0]	27.0 [23.0]	29.5 [33.0]	.928	<.001
Age at MTX initiation (y)	53.0 [20.0]	54.0 [19.0]	52.0 [22.0]	.344	53.0 [25.1]	52.1 [20.7]	53.1 [30.3]	.848	.359
Disease duration at MTX initiation (y)	0.04 [0.1]	0.04 [0.1]	0.05 [0.2]	.328	13.9 [18.4]	15.7 [20.2]	10.7 [16.7]	.093	<.001
BMI	27.5 [6.7]	27.3 [6.1]	28.1 [7.5]	.930	26.7 [7.5]	27.3 [5.5]	26.1 [6.5]	.175	.087
PASDAS	4.4 (1.1)	4.3 (1.2)	4.4 (1.1)	.402	-	-	-	-	-
SJC66	3.0 [5.0]	3.0 [4.0]	3.0 [4.3]	.014	-	-	-	-	-
TJC68	4.0 [6.0]	4.0 [6.0]	4.0 [7.0]	.044	-	-	-	-	-
Enthesitis (LEI)	0.0 [1.0]	0.0 [1.0]	0.0 [2.0]	<.001	-	-	-	-	-
Dactylitis count	0.0 [0.0]	0.0 [0.8]	0.0 [0.0]	.175	-	-	-	-	-
CRP	5.0 [11.0]	6.0 [10.4]	4.6 [10.6]	.055	-	-	-	-	-
HAQ	0.8 [0.7]	0.6 [0.8]	0.9 [0.6]	<.001	-	-	-	-	-
Physician Global VAS	27.0 [26.8]	29.0 [29.0]	24.0 [25.5]	.010	-	-	-	-	-
Patient Global VAS	47.0 [40.8]	43.0 [41.0]	51.0 [37.0]	.012	-	-	-	-	-
SF-36 PCS	39.2 (8.2)	40.8 (8.0)	37.5 (8.1)	<.001	-	-	-	-	-
PASI	2.0 [4.2]	2.5 [5.0]	1.0 [3.4]	<.001	7.3 [5.9]	6.9 [7.5]	7.8 [4.8]	.377	<.001
PsA diagnosis	-	-	-	-	21 (9.7%)	9 (7.0%)	12 (13.5%)	.112	-
Biologic naive	525 (100%)	262 (100%)	263 (100%)	-	209 (96.3%)	121 (94.5%)	88 (98.9%)	.092	<.001

BMI, body mass index; CRP, C-reactive protein; F, female; HAQ, Health Assessment Questionnaire; LEI, Leeds Enthesitis Index; M, male; MTX, methotrexate; PASDAS, Psoriatic Arthritis Disease Activity Score; PASI, Psoriasis Area and Severity Index; PsA, psoriatic arthritis; PsO, psoriasis; SF-36 PCS, Physical Component Summary Short Form 36; SJC66, swollen joint count of 66 joints; TJC68, Tender Joint Count of 68 Joints; VAS, Visual Analogue Scale.

Data are presented as n(%), median [IQR], mean (SD).

Percentages are calculated using the total number of available data for that characteristic per group.

Missing data psoriatic arthritis cohort: age at diagnosis 1.5%, disease duration 1.5%, BMI 10.4%, PASDAS 40.6%, SJC66 16.6%, TJC68 16.6%, LEI 16.0%, dactylitis count 16.2%, CRP 24.0%, HAQ 27.2%, Physician Global VAS 20.0%, Patient Global VAS 29.9%, SF-36 29.0%, and PASI 41.1%.

Missing data psoriasis cohort: age at diagnosis 4.6%, disease duration 4.6%, BMI 27.2%, and PASI 51.6%.

### Drug survival

In the PsA cohort, unadjusted Kaplan-Meier curves showed shorter drug survival in female compared with male patients, with a 3-year overall survival rate of 31.0% and 55.7%, respectively (*P* < .001) (Fig). In contrast, within the PsO cohort, shorter drug survival was observed for males compared with females (3-year overall survival 45.3% and 62.5%, respectively), although not statistically significant. Reasons for MTX discontinuation are presented in Supplementary Table S1. Similar patterns between males and females in both cohorts were observed when assessing treatment discontinuation due to ineffectiveness and adverse events specifically.

**Figure:**
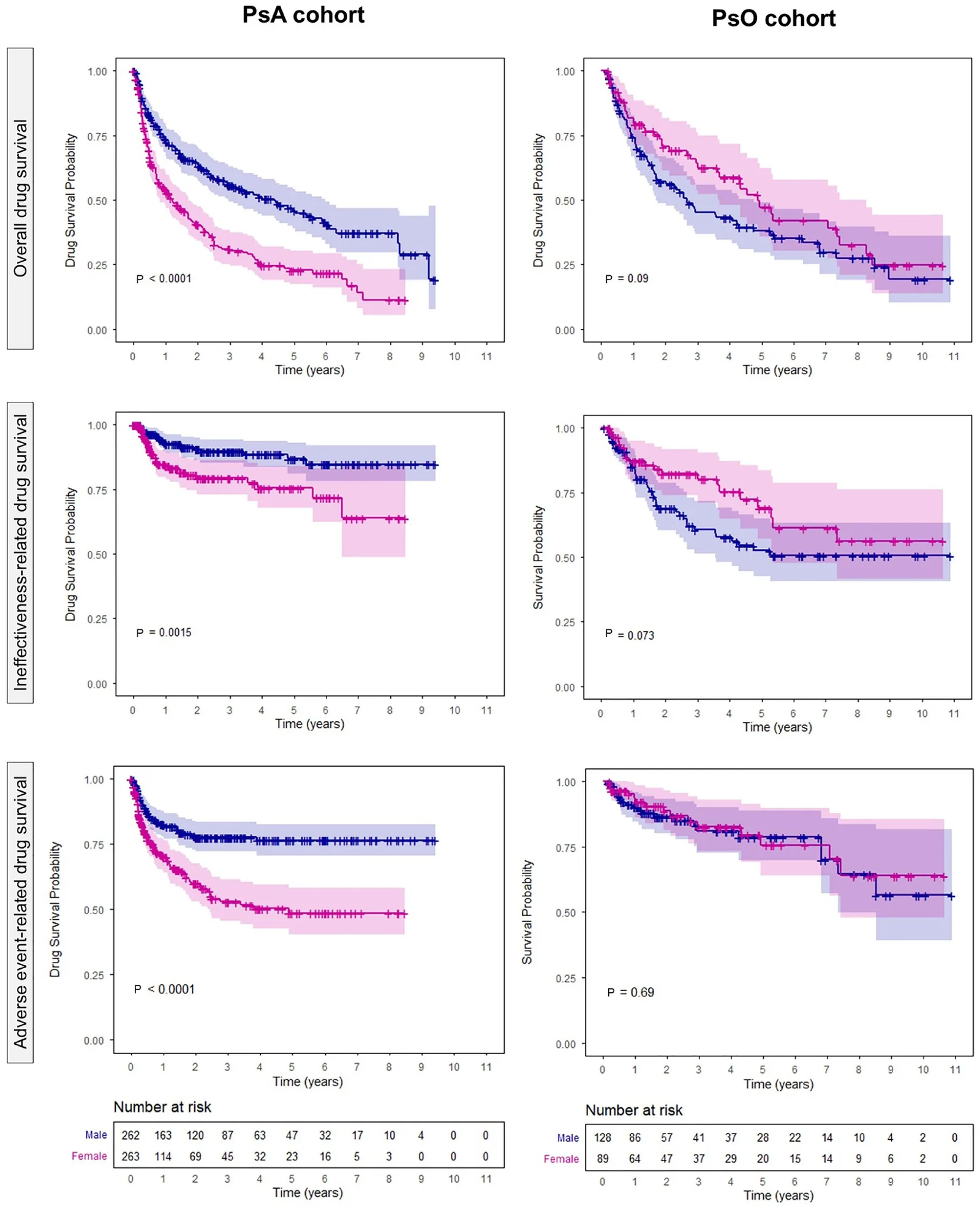
Kaplan-Meier curves comparing methotrexate drug survival in male (blue) compared with female patients (pink) in the psoriatic arthritis (PsA) or psoriasis (PsO) cohort.

Sensitivity analysis showed that patients with PsO and concomitant PsA did not impact MTX drug survival outcomes in the PsO cohort (Supplementary Fig S1).

Cox regression analyses adjusted for the precision variables age, disease duration, BMI, PsA diagnosis, and disease activity (Supplementary Table S2) confirmed the findings from the unadjusted analyses: among patients in the PsA cohort, female sex was associated with shorter overall drug survival (hazard ratio [HR] = 2.5; 95% CI, 1.6-4.1; *P* < .001) and drug survival related to ineffectiveness (HR = 3.1; 95% CI, 1.3-7.6; *P* = .013). Drug survival related to adverse events was not statistically significantly associated with sex in the PsA cohort (HR = 6.3; 95% CI, 0.7-53.6; *P* = .092). For patients in the PsO cohort, female sex was associated with longer overall drug survival (HR = 0.6; 95% CI, 0.4-0.9; *P* = .008) and ineffectiveness-related drug survival (HR = 0.4; 95% CI, 0.2-0.8; *P* = .009). Adverse event-related drug survival was also not statistically significantly associated with sex in the PsO cohort (HR = 0.6; 95% CI, 0.4-1.0; *P* = .074).

To investigate if sex-specific patterns of MTX drug survival might be influenced by different life stages, Kaplan-Meier curves were further stratified by young adults (≤30 years) and adults (>30 years) (Supplementary Fig S2). In both the PsA and the PsO cohort, young adult males had a lower overall drug survival compared with adult males (respectively, *P* = .010 and *P* < .001). In the PsO cohort, young adult males also had a significantly lower drug survival compared with young adult females (*P* = .03).

### MTX treatment regimens

In both the PsA and the PsO cohort, MTX was most frequently administered orally (respectively, 63.8% and 56.2%). When examining differences between males and females in the PsA cohort, we see that males more frequently used oral MTX (71.0% vs 56.7%, *P* < .001), whereas females more often used subcutaneous (19.2% vs 6.5%, *P* < .001). In addition, more female patients used both oral and subcutaneous MTX during their treatment episode (32.7% vs 20.6%, *P* = .002). A similar trend was observed in the PsO cohort (oral, M: 59.4% vs F: 51.6%, *P* = .256; subcutaneous, M: 9.4% vs F: 12.4%, *P* = .482; both oral and subcutaneous; M: 19.5% vs F: 32.6%, *P* = .029), although not all these differences were statistically significant.

The average dosing of MTX during the treatment episode was higher in the PsA compared with the PsO cohort (18.2 [7.5] mg/wk vs 15.0 [3.3] mg/wk, *P* < .001). However, the average dose did not differ between males and females in the PsA cohort (M:17.8 [7.4] mg/wk vs F: 18.6 [7.8] mg/wk, *P* = .733) and the PsO cohort (M:15.0 [2.6] mg/wk vs F 15.0 [4.3] mg/wk, *P* = .507). The maximum dosing of MTX during the treatment episode also did not differ between female and male patients in the PsA cohort (M: 25.0 [5.0] mg/wk vs F: 25.0 [10.0] mg/wk, *P* = .521) and the PsO cohort (M:15.0 [0.0] mg/wk vs F 15.0 [5.0] mg/wk, *P* = .257). The maximum dose was higher in the PsA cohort compared with the PsO cohort (25.0 [10.0] vs 15.0 [5.0], *P* = .257). MTX dosing at the moment of treatment discontinuation was similar for male and female patients in both the PsA cohort (M: 15.0 mg/wk [12.5] vs F: 15.0 mg/wk [10.0], *P* = .344) and the PsO cohort (M: 15.0 mg/wk [0.0] vs F: 15.0 mg/wk [6.3], *P* = .443).

### Disease severity at MTX discontinuation

Disease severity scores at the moment of MTX discontinuation are presented in Table 2. The PASDAS score was higher in female patients compared with male patients in the PsA cohort (M: 3.1 [1.5] vs F: 3.9 [1.5], *P* = .037). For patients in the PsO cohort, males had more severe disease compared with females, with PASI scores of 4.8 [6.4] and 3.5 [3.7], respectively (*P* = .032).

**Table 2:** Characteristics of patients in the psoriatic arthritis and psoriasis cohort at the moment of methotrexate discontinuation

	PsA cohort				PsO cohort			
	Total	Males	Females	*P* value (M vs F)	Total	Males	Females	*P* value (M vs F)
No. of patients	525	262	263	-	217	128	89	-
No. of patients who discontinued MTX	288 (53.3%)	120 (45.8%)	168 (63.9%)	<.001	118 (54.4%)	75 (58.6%)	43 (48.3 %)	.135
PASDAS	3.5 (1.3)	3.1 (1.5)	3.9 (1.0)	.037	-	-	-	-
SJC66	1.0 [3.0]	0.5 [3.8]	1.0 [2.8]	.0981	-	-	-	-
TJC68	3.0 [7.0]	2.0 [6.8]	3.0 [6.0]	.280	-	-	-	-
Enthesitis (LEI)	0.0 [1.0]	0.0 [0.0]	0.5 [1.8]	.005	-	-	-	-
Dactylitis count	0.0 [0.0]	0.0 [0.0]	0.0 [0.0]	.362	-	-	-	-
CRP	3.0 [7.2]	2.7 [4.0]	3.6 [8.0]	.163	-	-	-	-
HAQ	0.6 [1.0]	0.3 [0.8]	0.8 [0.8]	<.001	-	-	-	-
Physician Global VAS	13.0 [20.0]	13.0 [24.0]	13.0 [19.0]	.988	-	-	-	-
Patient Global VAS	34.5 [43.8]	24.0 [36.0]	47.0 [44.0]	.003	-	-	-	-
SF-36 PCS	40.5 (9.0)	44.8 (9.7)	37.5 (7.2)	<.001	-	-	-	-
PASI	0.6 [2.0]	0.8 [3.0]	0.2 [1.5]	.066	4.4 [4.2]	4.8 [6.4]	3.5 [3.7]	.032

CRP, C-reactive protein; F, female; HAQ, Health Assessment Questionnaire; LEI, Leeds Enthesitis Index; M, male; MTX, methotrexate; PASDAS, Psoriatic Arthritis Disease Activity Score; PASI, Psoriasis Area and Severity Index; PsA, psoriatic arthritis; PsO, psoriasis; SF-36 PCS, Physical Component Summary Short Form 36; SJC66: swollen joint count of 66 joints; TJC68, Tender Joint Count of 68 Joints; VAS, Visual Analogue Scale.

Data are presented as n(%), median [IQR], mean (SD).

Percentages are calculated using the total number of available data for that characteristic per group.

Missing data psoriatic arthritis cohort: PASDAS 81.3%, SJC66 66.7%, TJC68 66.7%, LEI 66.7%, dactylitis count 66.7%, CRP 69.8%, HAQ 72.2%, Physician Global VAS 67.0%, Patient Global VAS 73.6%, SF-36 68.8%, and PASI 75.0%.

Missing data psoriasis cohort: PASI 75.1%.

### Adverse events leading to MTX discontinuation in patients with PsO

In the PsO cohort, ‘gastrointestinal disorders’ (17.5% of the total number of MTX treatment episodes), ‘general disorders and administration site reactions’ (15.2%), and ‘investigations’ (ie, abnormal laboratory findings) (13.4%) were the most common reasons to discontinue MTX treatment (Supplementary Table S3). The most pronounced sex difference in adverse events leading to MTX discontinuation in the PsO cohort was observed for fatigue, with males discontinuing treatment due to fatigue more frequently than females (M: 8.6% vs F: 2.2%). A wish for a child or pregnancy was rarely (n = 2 males, n = 1 female) reported as a reason for MTX discontinuation.

### Subsequent treatment modality after MTX discontinuation

Of the patients within the PsA cohort who discontinued MTX treatment, there was no difference between sexes in the number of patients starting bDMARD therapy within 90 days following MTX discontinuation (M: 9.2% vs F: 10.7%, *P* = .678). Among patients within the PsO cohort, males more frequently initiated bDMARD therapy following MTX discontinuation compared with females (M: 52.1% vs F: 26.8%, *P* = .009), whereas females more often de-escalated therapy to topicals (M: 32.9% vs F: 53.7%, *P* = .031).

## DISCUSSION

This real-world registry study identified distinct sex-specific patterns in MTX drug survival among patients with PsA and PsO, underscoring that the influence of sex on MTX persistence differs by disease context. In the PsA cohort, MTX drug survival was shorter among females, whereas in the PsO cohort, male patients showed shorter drug survival. These differences were accompanied by sex-specific patterns in disease activity at the time of MTX discontinuation and in subsequent treatment escalation.

In the PsA cohort, females showed higher disease activity at the time of MTX discontinuation, whereas in the PsO cohort, higher disease activity was observed among males. Within the PsO cohort, male patients more often initiated bDMARDs as subsequent therapy compared with female patients.

The observation of a shorter drug survival among females in the PsA cohort aligns with existing literature. Previous studies across PsA, PsO, and other immune-mediated inflammatory diseases have consistently reported shorter drug survival of both conventional and biologic DMARDs in female patients [9,[12], [13], [14],[21], [22], [23]]. Females are more likely to discontinue therapy due to both inefficacy and adverse events [9,24,25]. In addition, sex differences in pharmacokinetics of MTX, including lower clearance in females, may also contribute to a higher risk of toxicity, thereby increasing the likelihood of treatment discontinuation [26]. These sex differences may not be fully explained by BMI alone, as BMI was comparable between females and males in our study. Instead, sex-specific differences in, for example, body composition may also play an important role [27]. Furthermore, analysis of the individual PASDAS components in the PsA cohort suggests that the higher disease activity observed in females was primarily driven by patient-reported measures. However, as several objective disease activity measures also showed numerically higher values in females, a contribution of objective disease activity cannot be excluded. Taken together, these mechanisms provide a biologically and clinically plausible explanation for the shorter MTX drug survival observed among females with PsA in the present study. In contrast, the finding of shorter MTX drug survival among males in the PsO cohort differs from previously published literature [15,28,29] and, to our knowledge, has not been reported before. This reversal of the expected sex pattern suggests that factors beyond sex-related biological differences may influence MTX persistence in PsO. However, it is also important to note that sex-related factors cannot be fully disentangled from gender-related factors, which are not assessed in this study, and may also influence treatment outcomes. In addition, disease-specific treatment trajectories and clinical context are likely to play an important role.

PsA and PsO are distinct diseases that differ in terms of disease course, timing of systemic therapy initiation, and clinical management. First, patients with PsO were diagnosed at a younger age and had longer disease duration before MTX initiation, often following topical or nonsystemic therapies. In contrast, patients with PsA commenced systemic treatment earlier after diagnosis. These differences may shape treatment expectations and persistence, with patients with PsO potentially more accepting of MTX despite adverse events. In addition, the clinical setting in which MTX is prescribed may differ, ultimately affecting drug survival outcomes. For instance, rheumatologists primarily focus on joint disease activity, whereas dermatologists prioritise skin involvement, which can influence decisions regarding continuation or discontinuation of MTX. This difference in approach may also explain why patients with PsA are, on average, prescribed higher MTX dosages.

Although younger age, especially among male patients in the PsO cohort, was associated with shorter MTX drug survival, family planning considerations are unlikely to fully account for the observed sex differences. Although MTX is contraindicated during pregnancy, and, in the past, MTX was also contraindicated for male patients during conception [19,30], a wish for a child or pregnancy was rarely reported as a reason for discontinuation. Therefore, other factors, such as treatment expectations or perceived disease burden, may be more relevant contributors to early discontinuation among young adults.

The strength of this study is the use of well-defined real-world prospective cohorts encompassing a large number of patients with PsA and PsO diagnosed by rheumatologists or dermatologists, respectively, with extended follow-up. However, a limitation of this study was the amount of missing data, especially for the patient characteristics at the moment of MTX discontinuation. As data were collected during routine clinical care, not all patients had measurements available within the predefined window around MTX discontinuation. Consequently, comparisons of patient characteristics at discontinuation are based on a relatively small subset of patients and should be interpreted with caution. In addition, more information on adverse events for the PsA cohort, as well as data on disease-related quality of life measures at the time of discontinuation, could have provided greater insight into the observed sex differences in drug survival.

In conclusion, this study demonstrates that sex and disease type significantly influence drug survival, disease activity, and adverse events in patients with PsA and PsO treated with MTX. While shorter MTX drug survival among females with PsA is consistent with existing literature, the shorter drug survival observed among males with PsO represents a novel finding which warrants further investigation. These findings underscore the importance of considering both sex- and disease-specific patterns to optimise and individualise MTX therapy in PsA and PsO.

## CRediT authorship contribution statement

**Liana Barenbrug:** Writing – original draft, Visualization, Project administration, Methodology, Formal analysis, Conceptualization. **Gonul Hazal Koc:** Writing – review & editing, Project administration, Data curation. **Marisol E Otero:** Writing – review & editing, Project administration, Data curation. **Emmerik F A Leijten:** Writing – review & editing, Supervision, Conceptualization. **Sander I van Leuven:** Writing – review & editing, Supervision, Conceptualization. **Irene E van der Horst-Bruinsma:** Writing – review & editing, Supervision, Conceptualization. **Juul M P A van den Reek:** Writing – review & editing, Supervision, Conceptualization. **Elke M G J de Jong:** Writing – review & editing, Supervision, Conceptualization. **Marc R Kok:** Writing – review & editing, Supervision, Conceptualization. **Marijn Vis:** Writing – review & editing, Supervision, Conceptualization. **Michelle L M Mulder:** Writing – review & editing, Supervision, Conceptualization.

## Competing interests

MEO has acted as a consultant for Eli Lilly. EFAL has received research grants from Eli Lilly and Novartis. IEvdH-B has acted as a paid speaker for Eli Lilly, BMS, AbbVie, Pfizer, MSD, and UCB and has received financial grants from Eli Lilly, MSD, Pfizer, AbbVie, and UCB. JMPAvdR carried out clinical trials for AbbVie, Celgene, Almirall, AstraZeneca, and Janssen; has received speaking fees/attended advisory boards from AbbVie, Janssen, Zoetis, BMS, Almirall, LEO Pharma, Novartis, UCB, and Lilly; has received reimbursement for attending or chairing a symposium from Janssen, Pfizer, Celgene, AbbVie, and EADV, and is a coordinator of the BioCAPTURE registry for which the institution has received funding from AbbVie, Janssen, Lilly, Almirall, Novartis, UCB, and LEO Pharma. All funding is not personal but goes to the independent research fund of the Department of Dermatology of Radboudumc Nijmegen, the Netherlands. EMGJdJ received research grants for the Department of Dermatology of the Radboud University Medical Center Nijmegen, the Netherlands from AbbVie, Almirall, J&J, LEO Pharma, Lilly, and UCB; and acted as consultant and/or paid speaker for and/or participated in research sponsored by companies that manufacture drugs used for the treatment of psoriasis including AbbVie, Almirall, Boehringer-Ingelheim, Bristol Myers Squibb, J&J, LEO Pharma, Lilly, and UCB. All funding is not personal but goes to the Institution Radboud University Medical Center Nijmegen, the Netherlands. MRK received research grants from the Department of Rheumatology and Clinical Immunology from Janssen. MLMM has received speaker fees and compensation for participation in advisory boards from Novartis. All other authors declare they have no competing interests.

## Appendix Supplementary materials

Image, application 1

## Footnotes

Supplementary material associated with this article can be found in the online version at doi:10.1016/j.ero.2026.100255.
