# Supplementary material for: Sex-specific outcomes in methotrexate therapy among patients with psoriatic arthritis and psoriasis
Source: EULAR Rheumatol Open. 2026 Oct 10;2(4):100255. doi: 10.1016/j.ero.2026.100255 (PMC13678078; doi:10.1016/j.ero.2026.100255)
Supplement: Supplementary file 1 [file mmc1.docx]

Supplementary material

**Supplementary Figure S1| Kaplan-Meier curves comparing methotrexate drug survival in male (blue) compared to female (pink) patients within the psoriasis cohort without concomitant psoriatic arthritis.**

**Supplementary Figure S2| Kaplan-Meier survival curves comparing methotrexate drug survival in male compared to female patients in the psoriatic arthritis (PsA) and psoriasis (PsO) cohort stratified for young adults (≤ 30 years) and adults (>30 years).**

**Supplementary Table 1| Reasons for methotrexate discontinuation for males and females in the psoriatic arthritis and psoriasis cohort.**

|  | **PsA cohort** | | | **PsO cohort** | | |
| --- | --- | --- | --- | --- | --- | --- |
|  | **Total** | **Males** | **Females** | **Total** | **Males** | **Females** |
| **Adverse events** | 127 (24.2%) | 45 (17.2%) | 82 (31.2%) | 37 (17.1%) | 23 (18.0%) | 14 (15.7%) |
| **Ineffectiveness** | 46 (8.8%) | 17 (6.5%) | 29 (11.0%) | 21 (9.7%) | 15 (11.7%) | 6 (6.7%) |
| **Adverse events and ineffectiveness** | 14 (2.7%) | 5 (1.9%) | 9 (3.4%) | 34 (15.7%) | 22 (17.2%) | 12 (13.5%) |
| **Remission** | 43 (8.2%) | 24 (9.2%) | 19 (7.2%) | 6 (2.8%) | 3 (2.3%) | 3 (3.4%) |
| **Deceased** | 1 (0.2%) | 1 (0.4%) | 0 (0%) | 4 (1.8%) | 2 (1.6%) | 2 (2.2%) |
| **Other** | 49 (9.3%) | 25 (29.5%) | 24 (9.1%) | 12 (5.5%) | 8 (6.3%) | 4 (4.5%) |
| **Family planning / pregnancy*** | - | - | - | 3 (1.4%) | 2 (1.6%) | 1 (1.2%) |
| **Unknown** | 8 (1.5%) | 3 (1.1%) | 5 (1.9%) | - | - | - |
| **Toal number of patients discontinuing MTX** | 288 (54.9%) | 120 (45.8%) | 168 (63.8%) | 117 (53.9%) | 75 (58.6%) | 42 (47.2%) |
| **Total number of patients** | 525 | 262 | 263 | 217 | 128 | 89 |

*Data is presented as n(%).*

*Percentages are calculated with respect to the total number of patients in the group of interest.*

**Family planning/pregnancy was not specifically assessed as reason of discontinuation in the DEPAR registry.*

| **Supplementary Table S2\| Multivariable Cox regression analyses assessing the effect of sex on methotrexate drug discontinuation in patients with psoriatic arthritis and psoriasis being.** | | | | | | | | | | |
| --- | --- | --- | --- | --- | --- | --- | --- | --- | --- | --- |
|  | **PsA cohort** | | | | | **PsO cohort** | | | | |
| **Variable** | **HR** | **95% CI** | | | **p-value** | **HR** | **95% CI** | | | **p-value** |
| *Overall drug survival* | | | | | | | | | | |
| **Sex (ref = male)** | **2.533** | | **1.559** | **4.115** | **<0.001** | **0.573** | **0.380** | **0.865** | **0.008** | |
| Age at start MTX (years) | 0.985 | | 0.969 | 1.001 | 0.068 | 0.953 | 0.938 | 0.967 | <0.001 | |
| Disease duration until start of MTX (years) | 0.932 | | 0.424 | 2.048 | 0.861 | 1.019 | 1.001 | 1.038 | 0.037 | |
| Baseline BMI | 0.980 | | 0.935 | 1.027 | 0.403 | 1.002 | 0.957 | 1.049 | 0.921 | |
| Psoriatic arthritis (ref = yes) | - | | - | - | - | 0.800 | 0.431 | 1.485 | 0.480 | |
| Baseline PASDAS | 0.804 | | 0.650 | 0.994 | 0.804 | - | - | - | - | |
| Baseline PASI | - | | - | - | - | 0.976 | 0.9381 | 1.023 | 0.307 | |
| *Ineffectiveness related drug survival* | | | | | | | | | | |
| **Sex (ref= male)** | **3.108** | | **1.267** | **7.626** | **0.013** | **0.445** | **0.242** | **0.818** | **0.009** | |
| Age at start biologic (years) | 0.984 | | 0.958 | 1.012 | 0.267 | 0.939 | 0.918 | 0.961 | <0.001 | |
| Disease duration until start of biologic (years) | 1.042 | | 0.320 | 3.385 | 1.042 | 1.019 | 0.992 | 1.048 | 0.169 | |
| Baseline BMI | 1.005 | | 0.930 | 1.087 | 0.894 | 1.008 | 0.944 | 1.076 | 0.811 | |
| Psoriatic arthritis (ref = yes) | - | | - | - | - | 0.904 | 0.0.345 | 2.367 | 0.838 | |
| Baseline PASDAS | 1.038 | | 0.706 | 1.527 | 0.037 | - | - | - | - | |
| Baseline PASI | - | | - | - | - | 0.992 | 0.923 | 1.065 | 0.819 | |
| *Adverse event related drug survival* | | | | | | | | | | |
| **Sex (ref = male)** | **6.311** | | **0.743** | **53.641** | **0.092** | **0.618** | **0.365** | **1.048** | **0.074** | |
| Age at start MTX (years) | 0.942 | | 0.892 | 0.995 | 0.031 | 0.943 | 0.924 | 0.963 | <0.001 | |
| Disease duration until start of MTX (years) | 0.676 | | 0.014 | 31.494 | 0.842 | 1.029 | 1.004 | 1.055 | 0.022 | |
| Baseline BMI | 1.029 | | 0.906 | 1.168 | 0.665 | 0.998 | 0.936 | 1.064 | 0.951 | |
| Psoriatic arthritis (ref = yes) | - | | - | - | - | 0.817 | 0.373 | 1.788 | 0.612 | |
| Baseline PASDAS | 0.953 | | 0.449 | 2.021 | 0.900 | - | - | - | - | |
| Baseline PASI | - | | - | - | - | 0.974 | 0.919 | 1.032 | 0.327 | |

*PsA, psoriatic arthritis; PsO, psoriasis; HR, Hazard Ratio; CI; Confidence Interval; MTX, methotrexate; BMI, body mass index; PASDAS, Psoriatic Arthritis Disease Activity Score; PASI, Psoriasis Area and Severity Index.*

| **Supplementary Table S3\| Adverse events leading to methotrexate discontinuation in patients within the psoriasis cohort** | | | | | | |
| --- | --- | --- | --- | --- | --- | --- |
| **Side effect** | **Total** | **%** | **Males** | **%** | **Females** | **%** |
| **Total number of treatment episodes** | **217** |  | **128** |  | **89** |  |
| **Total number of treatment episodes discontinued due to adverse events** | **71** | **32.7** | **45** | **35.2** | **26** | **29.2** |
| **Blood and lymphatic disorders** | **3** | **1,4** | **0** | **0,0** | **3** | **3,4** |
| Gingival bleeding | 1 | 0,5 | 0 | 0,0 | 1 | 1,1 |
| Iron deficiency anaemia | 1 | 0,5 | 0 | 0,0 | 1 | 1,1 |
| Lymphadenopathy | 1 | 0,5 | 0 | 0,0 | 1 | 1,1 |
| **Cardiac disorders** | **1** | **0,5** | **0** | **0,0** | **1** | **1,1** |
| Palpitations | 1 | 0,5 | 0 | 0,0 | 1 | 1,1 |
| **Ear and labyrinth disorders** | **1** | **0,5** | **0** | **0,0** | **1** | **1,1** |
| Tinnitus | 1 | 0,5 | 0 | 0,0 | 1 | 1,1 |
| **Gastrointestinal disorders** | **38** | **17,5** | **22** | **17,2** | **16** | **18,0** |
| Nausea | 25 | 11,5 | 13 | 10,2 | 12 | 13,5 |
| Gastro-intestinal complaints, not specified | 5 | 2,3 | 3 | 2,3 | 2 | 2,2 |
| Vomiting | 5 | 2,3 | 4 | 3,1 | 1 | 1,1 |
| Diarrhoea | 3 | 1,4 | 2 | 1,6 | 1 | 1,1 |
| **General disorders and administration site reactions** | **33** | **15,2** | **21** | **16,4** | **12** | **13,5** |
| Fatigue | 13 | 6,0 | 11 | 8,6 | 2 | 2,2 |
| Malaise | 8 | 3,7 | 5 | 3,9 | 3 | 3,4 |
| Influenza like illness | 7 | 3,2 | 4 | 3,1 | 3 | 3,4 |
| Decreased appetite | 2 | 0,9 | 1 | 0,8 | 1 | 1,1 |
| Injection site bruising | 2 | 0,9 | 0 | 0,0 | 2 | 2,2 |
| Hot flush | 1 | 0,5 | 0 | 0,0 | 1 | 1,1 |
| **Injury poisoning and procedural complications** | **1** | **0,5** | **0** | **0,0** | **1** | **1,1** |
| Fall | 1 | 0,5 | 0 | 0,0 | 1 | 1,1 |
| **Infections and manifestations** | **3** | **1,4** | **1** | **0,8** | **2** | **2,2** |
| Skin infection | 2 | 0,9 | 1 | 0,8 | 1 | 1,1 |
| Lung abscess | 1 | 0,5 | 0 | 0,0 | 1 | 1,1 |
| **Investigations** | **29** | **13,4** | **20** | **15,6** | **9** | **10,1** |
| Hepatic enzyme abnormal | 22 | 10,1 | 14 | 10,9 | 8 | 9,0 |
| Lymphocyte count decreased | 3 | 1,4 | 3 | 2,3 | 0 | 0,0 |
| Platelet count decreased | 2 | 0,9 | 1 | 0,8 | 1 | 1,1 |
| White blood cell count decreased | 2 | 0,9 | 2 | 1,6 | 0 | 0,0 |
| **Musculoskeletal and connective tissue disorder** | **3** | **1,4** | **3** | **2,3** | **0** | **0,0** |
| Myalgia | 2 | 0,9 | 2 | 1,6 | 0 | 0,0 |
| Arthralgia | 1 | 0,5 | 1 | 0,8 | 0 | 0,0 |
| **Nervous system disorders** | **11** | **5,1** | **10** | **7,8** | **1** | **1,1** |
| Headache | 5 | 2,3 | 4 | 3,1 | 1 | 1,1 |
| Dizziness | 3 | 1,4 | 3 | 2,3 | 0 | 0,0 |
| Anosmia | 1 | 0,5 | 1 | 0,8 | 0 | 0,0 |
| Dysgeusia | 1 | 0,5 | 1 | 0,8 | 0 | 0,0 |
| Neuropathy peripheral | 1 | 0,5 | 1 | 0,8 | 0 | 0,0 |
| **Psychiatric disorders** | **1** | **0,5** | **1** | **0,8** | **0** | **0,0** |
| Libido decreased | 1 | 0,5 | 1 | 0,8 | 0 | 0,0 |
| **Respiratory, thoracic and mediastinal disorders** | **3** | **1,4** | **2** | **1,6** | **1** | **1,1** |
| Dyspnoea | 2 | 0,9 | 1 | 0,8 | 1 | 1,1 |
| Pneumonitis | 1 | 0,5 | 1 | 0,8 | 0 | 0,0 |
| **Renal and urinary disorders** | **1** | **0,5** | **1** | **0,8** | **0** | **0,0** |
| Polyuria | 1 | 0,5 | 1 | 0,8 | 0 | 0,0 |
| **Skin and subcutaneous tissue disorders** | **2** | **0,9** | **0** | **0,0** | **2** | **2,2** |
| Acne | 1 | 0,5 | 0 | 0,0 | 1 | 1,1 |
| Alopecia | 1 | 0,5 | 0 | 0,0 | 1 | 1,1 |
| **Total number of adverse events leading to methotrexate discontinuation** | **130** |  | **81** |  | **49** |  |

*Percentages are calculated with respect to the total number of treatment episodes of the group.*
